# Supplementary material for: Erucin Exerts Cardioprotective Effects on Ischemia/Reperfusion Injury through the Modulation of mitoKATP Channels
Source: Biomedicines. 2023 Dec 12;11(12):3281. doi: 10.3390/biomedicines11123281 (PMC10740937; doi:10.3390/biomedicines11123281)
Supplement: Supplementary file 1 [file biomedicines-11-03281-s001.zip › biomedicines-2738143-supplementary.pdf]

Supporting Information

Erucin Exerts Cardioprotective Effects on Ischemia/Reperfusion Injury through the Modulation of mitoKATP Channels

Lorenzo Flori [1], Rosangela Montanaro [2], Eleonora Pagnotta [3], Luisa Ugolini [3], Laura Righetti [3], Alma Martelli [1,4,5], Lorenzo Di Cesare Mannelli [6], Carla Ghelardini [6], Vincenzo Brancaleone [2], Lara Testai [1,4,5,*] and Vincenzo Calderone [1,4,5]

Department of Pharmacy, University of Pisa, 56120-Pisa, Italy; lorenzo.flori@farm.unipi.it; alma.martelli@unipi.it; lara.testai@unipi.it; vincenzo.calderone@unipi.it.

2 Department of Science, University of Basilicata, 85100, Potenza, Italy; vincenzo.brancaleone@unibas.it; rosangela.montanaro@unibas.it.

3 CREA-Council for Agricultural Research and Economics, Research Centre for Cereal and Industrial Crops, via di Corticella 133, 40128 Bologna, Italy; eleonora.pagnotta@crea.gov.it; luisa.ugolini@crea.gov.it; laura.righetti@crea.gov.it.

4 Interdepartmental Research Center Nutrafood "Nutraceuticals and Food for Health", University of Pisa, Pisa, Italy.

5 Interdepartmental Research Centre of Ageing Biology and Pathology, University of Pisa, Pisa, Italy

6 Department of Neuroscience, Psychology, Drug Research and Child Health–Neurofarba–Pharmacology and Toxicology Section, University of Florence, Florence, Italy; lorenzo.mannelli@unifi.it; carla.ghelardini@unifi.it

* Correspondence: lara.testai@unipi.it (L.T.)

Figure S1. Time-course recording of the fluorescence emitted after the addiction of thallium ion into suspended cardiac mitochondria.

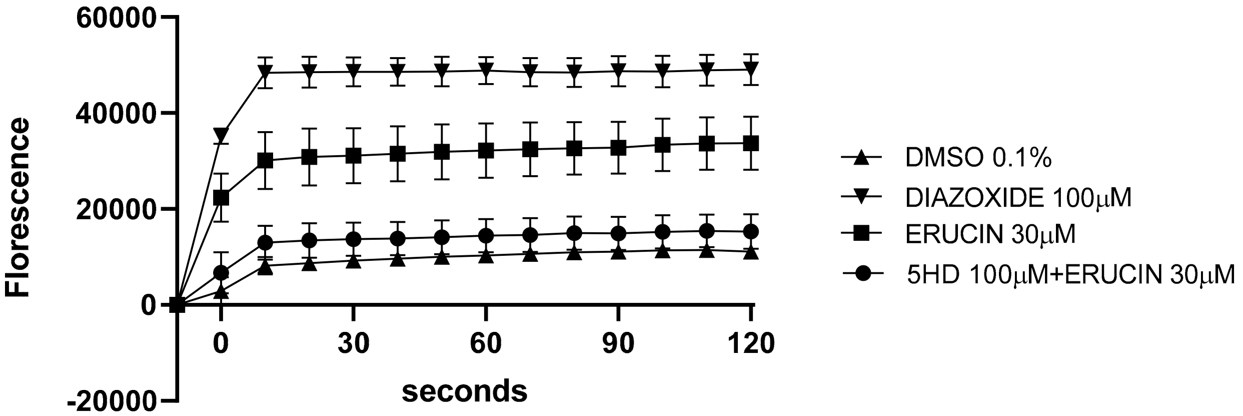

The increase in fluorescence (due to the entry of Tl+ into the matrix, through the potassium channels) was monitored for 120 seconds by EnSpire multi-plate reader; then the area under curve (AUC) was calculated for different treatments and subtracted the AUC obtained with vehicle. Diazoxide is considered as a positive control for the activation of mitoKATP channel, and established equal to 100%. The florescence emitted after treatment with erucin 30 microM or erucin 30 microM+5HD 100 microM was expressed as percentage of diazoxide.
